# Hematopoietic stem cells and betaherpesvirus latency

**DOI:** 10.3389/fcimb.2023.1189805

**Published:** 2023-06-06

**Authors:** Lindsey B. Crawford

**Affiliations:** ^1^Department of Biochemistry, University of Nebraska-Lincoln, Lincoln, NE, United States; ^2^Nebraska Center for Virology, University of Nebraska-Lincoln, Lincoln, NE, United States; ^3^Nebraska Center for Integrated Biomolecular Communication, University of Nebraska-Lincoln, Lincoln, NE, United States

**Keywords:** latency, betaherpesvirus, HCMV, HHV-5, HHV-6, HHV-7, hematopoiesis, hematopoietic stem/progenitor cell

## Abstract

The human betaherpesviruses including human cytomegalovirus (HCMV), human herpesvirus (HHV)-6a and HHV-6b, and HHV-7 infect and establish latency in CD34+ hematopoietic stem and progenitor cells (HPCs). The diverse repertoire of HPCs in humans and the complex interactions between these viruses and host HPCs regulate the viral lifecycle, including latency. Precise manipulation of host and viral factors contribute to preferential maintenance of the viral genome, increased host cell survival, and specific manipulation of the cellular environment including suppression of neighboring cells and immune control. The dynamic control of these processes by the virus regulate inter- and intra-host signals critical to the establishment of chronic infection. Regulation occurs through direct viral protein interactions and cellular signaling, miRNA regulation, and viral mimics of cellular receptors and ligands, all leading to control of cell proliferation, survival, and differentiation. Hematopoietic stem cells have unique biological properties and the tandem control of virus and host make this a unique environment for chronic herpesvirus infection in the bone marrow. This review highlights the elegant complexities of the betaherpesvirus latency and HPC virus-host interactions.

## 1 Introduction

**Figure 1:**
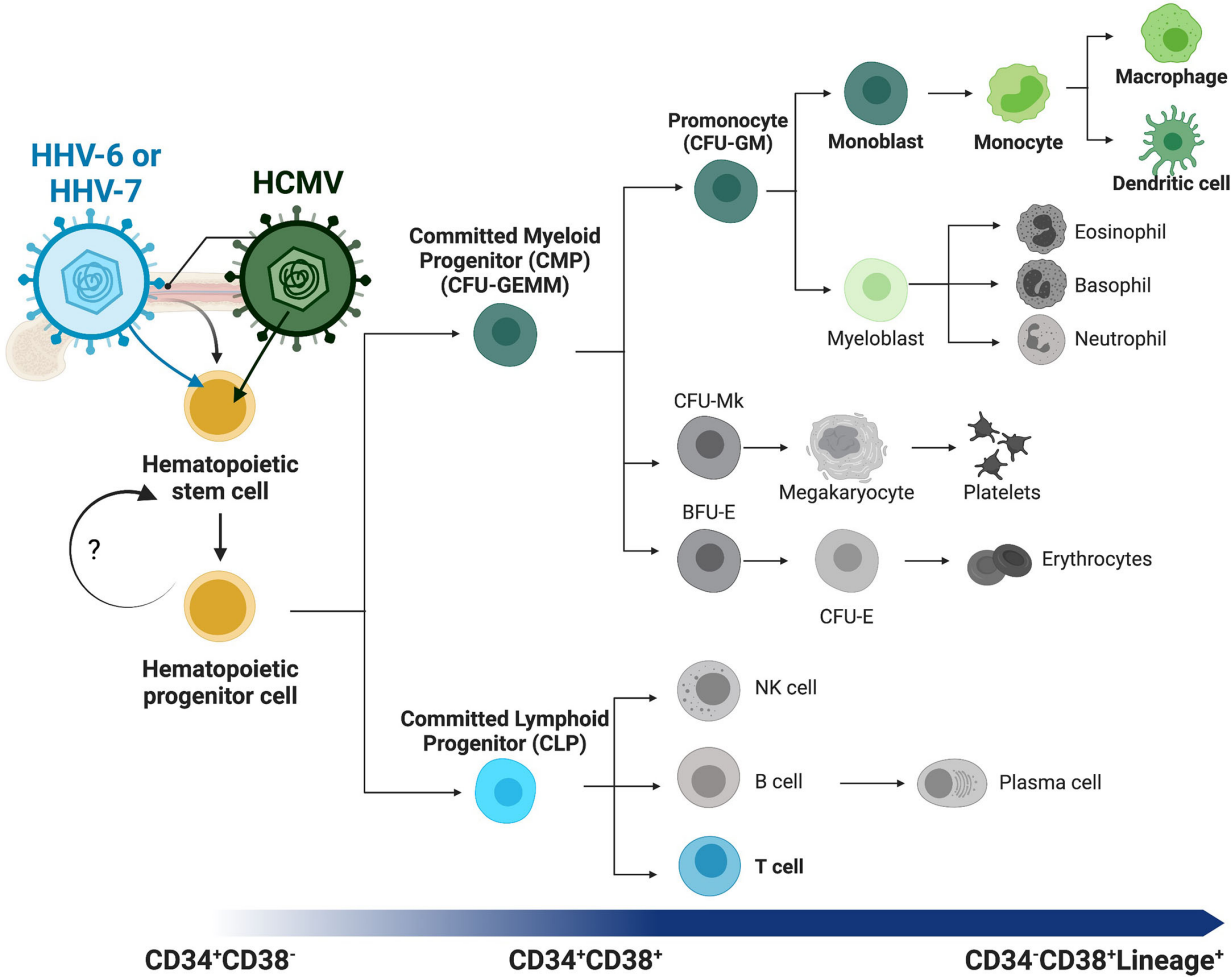
Overview of human hematopoiesis. Schematic overview of human hematopoiesis from early hematopoietic stem cells (HSCs) to more mature hematopoietic progenitors (HPCs), both populations with self-renewal and multilineage differentiation potential. The heterogeneous HPC population subsequently gives rise to a series of intermediate progenitors, including commitment to either the lymphoid or myeloid lineages through the committed lymphoid (CLP) or committed myeloid progenitor (CMP), respectively. Lineage analysis and differentiation capability of the CMP population and direct descendants can be assessed with the classic colony forming unit (CFU) assay, and populations distinguished by morphology and other characteristics. Lineage commitment of the monocytic lineage begins with the CFU-GM (CFU-granulocyte/macrophage) diverging from the CFU-Mk (CFU-megakaryocyte) and erythroid [BFU-E (burst-forming unit-erythroid) and CFU-E (CFU-erythroid)] lineages. These intermediate progenitors have more restricted self-renewal capacity and differentiate into mature and functional immune cells (i.e., monocytes, T-cells, B-cells, dendritic cells). Lineage tracing can be performed using cell surface receptors, beginning with CD34^+^CD38^-^ early HSCs and HPCs, maturation to committed progenitors, then finally mature immune cells that express lineage markers (Lineage+, e.g. CD3^+^ T-cells or CD14^+^ monocytes) and lack CD34 expression. Betaherpesviruses infect HPCs and specifically control differentiation to virus-favorable lineages. Highlighted (in color and bold) here with their lineage preferences are HHV-6 or HHV-7 to T-cell differentiation (blue) and HHV-5 (HCMV) to myeloid differentiation (green).

Hematopoiesis is a highly regulated, hierarchical, and multilinear process wherein stem cells differentiate into mature hematopoietic cells. Successful hematopoiesis is required for the formation and continual replenishment of all cellular components and maintenance of a functional immune system. This complex process is now believed to be partially reversable allowing dynamic control of stem cell fate and differentiation. The classic model of hematopoietic differentiation proposes that pluripotent hematopoietic stem cells (HSCs) are part of a larger pool of progenitors (HPCs) whose functions and abilities (including differentiation to specific lineages) vary depending on their specific characteristics including developmental status (e.g., fetal *vs* adult) and physical location (e.g., tissues *vs* bone marrow). In adults, HSCs reside in the bone marrow and differentiate through a series of progenitors (including the heterogeneous HPC population) which subsequently give rise to committed progenitors which, in turn, differentiate specifically into mature immune cells (**Figure 1***, overview*). These differentiation stages, the fate of the progenitor cells, and hematopoietic programs are controlled by a complex interplay between stem cells, the bone marrow niche, neighboring immune cells, and external and/or environmental factors (King et al., 2020).

In stem cells, progenitors, and mature blood lineage cells, the fundamental processes of self-renewal, quiescence, apoptosis, proliferation, and differentiation are governed by these interactions. Since hematopoiesis is an essential process for life, it makes sense that many processes required for maintenance are conserved. Under normal physiologic conditions, hematopoietic homeostasis is maintained in hematopoietic stem/progenitor cells (HS/HPCs) by a delicate, and complex, balance between all fundamental stem cell processes: self-renewal and/or apoptosis with proliferation and/or differentiation. Under stress conditions, including viral infection, fewer HS/HPCs undergo apoptosis while increased levels of cytokines and growth factors enhance proliferation and differentiation in order to repopulate and support immune system function. In healthy individuals, the hematopoietic system returns to baseline after stress conditions end. However, deregulation of hematopoietic conditions is associated with stress (Zhao and Baltimore, 2015) and ageing (Groarke and Young, 2019) as well as numerous disease states including cancer, autoimmune disorders, and chronic viral infection. Many viruses are associated with dysregulation of bone marrow function and pathogenic outcomes [reviewed (Kolb-Mäurer and Goebel, 2003; Pascutti et al., 2016)], and previous studies demonstrated direct productive and/or latent viral infection in HS/HPCs from diverse viral families, including retroviruses (Banerjee et al., 2010), parvovirus (Segovia et al., 2003), JC virus (Monaco et al., 1996), hepatitis C (Sansonno et al., 1998), measles (Manchester et al., 2002), and herpesviruses (Maciejewski et al., 1992; Isomura et al., 1997; Mirandola et al., 2000; Wu et al., 2006). This review discusses the evidence for betaherpesviruses infection of, and latency establishment in, HS/HPCs, and how the interactions between virus and host control hematopoietic programs.

## 2 Hematopoietic complexity: what defines an HSC?

HSCs are pluripotent stem cells that can differentiate into and generate all hemato-lymphoid lineage cells. The most widely used operational definition of a ‘classic HSC’ is characterization by expression of the surface receptor CD34, since the development of the sialomucin CD34 monoclonal antibody in the mid-1980s (Civin et al., 1984). However, the identity of an HSC is still somewhat unclear. First, CD34 expression is heterogeneous (Krause et al., 1996) and includes differentiating cells, committed progenitors and early multipotent progenitors as well as stem cells. Second, newer research suggests that the very earliest HSCs may lack CD34 expression (Sonoda, 2021). Third, and ironically, the function of CD34 on these cells is still not defined (AbuSamra et al., 2017). Regardless, CD34 expression is the classic and well accepted identifier for both HSCs and the slightly more mature HPC population, although different cells in these subsets likely have different functions. The ‘standard’ HS/HPC pool is currently defined by using a combination of CD34 expression (CD34^+^) and the absence of mature lineage markers (Lin-).

Functionally, a cell must meet four basic requirements to be an HSC: 1) the capability for self-renewal, 2) the capability to undergo apoptosis, 3) the maintenance of multilineage hematopoiesis, and 4) mobilization out of the bone marrow into the circulating blood. The most stringent test to evaluate if a population contains true HS/HPCs is pairing identity (surface marker expression) with function. For example, HSCs can be isolated *in vitro* using the surface receptor CD34 (Hao et al., 1996) and reconstitute irradiated recipients (Baum et al., 1992). Numerous studies and clinical applications provide support for human hematopoietic reconstitution using CD34^+^ cells for both autologous and allogeneic transplants (Weissman and Shizuru, 2008). In 2020, more than 20,000 hematopoietic cell transplants were performed in the United States alone (Health Resources and Services Administration, 2022) as therapy for diverse diseases including cancer, immunodeficiencies, autoimmune and blood disorders, and a variety of genetic conditions. However, many more individuals do not receive this well-established therapy due to a lack of appropriate donor cells and complications with donor quality. Of those that do, even well-matched transplant material of sufficient cells with good function can lead to rejection as a result of graft *vs* host disease and/or suppressed cell engraftment or immune function due to infectious disease complications. Human betaherpesviruses play a significant role in the success, or lack thereof, of hematopoietic stem cell transplant (Ljungman, 2010; Yoshikawa, 2018).

## 3 Human hematopoietic hierarchy and function

Long-term transplantation experiments, in a diversity of species, suggest a clonal diversity model where the HSC compartment consists of a fixed number of different types of HSCs, each with a preprogrammed fate (Muller-Siebrug et al., 2002; Lu et al., 2019; Teets et al., 2020). This coincides with the classic model of hematopoiesis where HS/HPCs maintain the stem cell compartment through regulated quiescence and self-renewal balanced with controlled apoptosis, proliferation, and differentiation as needed. These cells subsequently give rise to either the committed myeloid progenitor (CMP) or committed lymphoid progenitor (CLP) which, while still multipotent, have more restricted self-renewal capacity. These intermediates then differentiate specifically into mature (lineage-committed) immune cells (i.e. monocytes, T-cells, B-cells, dendritic cells) (**Figure 1**). Specific differentiation stages, progenitor cell fate, and hematopoiesis, are all controlled by complex interactions between hematopoietic cells and their environment, and by the fine-tuned control of hematopoietic viruses. Understanding the factors governing human hematopoiesis is essential to understand the mechanisms of viral biology and to specifically target or clear viral infections residing in these cells.

Further refinement of the identity and function of the HS/HPC pool is a first step in refining our knowledge of human hematopoiesis. Prior work pairing cell surface markers with functional studies identified different sub-populations enriched for HSC functions. For example, preferential long-term engraftment abilities are enriched in the CD34^+^CD38^-^ population (Steidl et al., 2002; Ishikawa et al., 2003; Venezia et al., 2004; Forsberg et al., 2005). Yet further refinement has proved elusive. While the surface marker ‘identity’ of these cells and the basic definition of a human HSC *vs* HPC are broadly defined (Parekh and Crooks, 2013), the functional ability of these cells to engraft varies. Prior work suggests a difference in cell type required for either short- or long-term reconstitution, and yet separation of early progenitors using additional markers (e.g. CD90) into these populations [putative early progenitors (CD38^-^CD90^+^) and short-term progenitors (CD38^+^CD90^-^)] shows that both support early engraftment and yet still have heterogeneous phenotypes (Cheung et al., 2012), meaning the true definition of an HSC *vs* an HPC and their different functions are still under-defined. This is key to understanding the biology of viruses, including the betaherpsvirues, which utilize the unique biology of these cells to establish latency as discussed below.

Recent work capitalizing on single cell genomics and proteomics has provided maps of hematopoietic commitment and differentiation, and characterized rare subpopulations (Triana et al., 2021) including with age- and tissue-related specifics (Andersson et al., 2014; Hennrich et al., 2018). Yet, the conclusions drawn from these studies have yet to come to a consensus, with some in support of the traditional hierarchical model (Pellin et al., 2019), some supporting a model with a limited number of defined “primed states” (Zheng et al., 2018), or the idea that hematopoietic commitment is a continuous process rather than discrete stages (Velten et al., 2017).

Combining these newer technologies with reconstitution models will refine our knowledge of *functional* hematopoietic populations. Functional analysis in humanized mouse models demonstrates the success rate of different human HS/HPC populations using serial transplants in immunodeficient mice and measurement of reconstitution. Since the early 1990s, these models have given us new insights into human hematopoiesis (Baum et al., 1992; Lapidot et al., 1992; Bock, 1997). Despite this, many putative HS/HPC populations lack full lineage reconstitution, likely due in part to the species mismatch and the lack of a fully supportive bone marrow microenvironment (Abarrategi et al., 2018; Stripecke et al., 2020; Martinov et al., 2021), and therefore a fully refined population for reconstitution has yet to be defined. Other differences even within a single species, including sex (Cui et al., 2022), age (Groarke and Young, 2019), and environment (i.e. inflammation (King et al., 2020), exert significant effects on stem cells, differentiation, and the risk for related malignancies; clearly demonstrating that while the basic principles are conserved, specific differences influence cell fate and function in hematopoiesis.

Viral infection also highlights these specific outcomes, as many viruses have evolved to manipulate hematopoiesis and specifically drive differentiation towards a virus-favorable outcome. Uniquely, however, different viruses manipulate different cellular pathways to specifically drive differentiation (**Figure 1***, highlighted lineages for beta-herpesviruses*). For example, common viruses that establish long-term (latent or chronic) infections, including retroviruses and herpesviruses, all infect HPCs but control hematopoiesis, drive differentiation to specific lineages, and manipulate specific cellular pathways in virus-specific manners (Banerjee et al., 2010; Stanojevic et al., 2022). Herpesvirus infection, in particular, is a common concern during hematopoietic cell (and solid organ) transplant, where immunosuppression provides a cellular environment promoting viral infection and/or reactivation, which in turn can lead to additional myelosuppression, which can ultimately result in graft failure (Ivana et al., 2022). Yet, while many mammalian species, from elephants to mice, are infected with herpesviruses, the evolutionary differences that prevent cross-infection, even between highly related primate betaherpesvirus species, highlight specific adaptations to the host immune system and distinct mechanisms of viral behavior (Cagliani et al., 2020; Fisher and Lloyd, 2020). Understanding these mechanisms will provide novel insights into viral infection (and treatment), immune function, and hematopoietic mechanisms.

## 4 Human betaherpesviruses

The human *Herpesviridae* (HHV) family is composed of large double-stranded DNA viruses in three subfamilies containing nine known distinct viruses. The alphaherpesviruses include HHV-1 [Herpes simplex virus (HSV)-1], HHV-2 (HSV-2), and HHV-3 [Varicella zoster virus (VZV)]. The betaherpesviruses include HHV-5 [human cytomegalovirus (HCMV)] and the *Roseloviruses*, including HHV-6a, HHV-6b, and HHV-7. The gammaherpesviruses include HHV-4 [Epstein-Barr virus (EBV)] and HHV-8 (Kaposi sarcoma-associated virus (KSHV)]. All members have a restricted host range, and unique infection, replication, and latency patterns. The differences in cell tropism and complex lifecycle stages highlight the unique properties of herpesviruses-cell host interactions.

Betaherpesviruses are ubiquitous and establish lifelong infections in the host. Infections typically occur early in life, with seroprevalence increasing with age and varying depending on geographical location and socioeconomic factors. Global seroprevalence of HCMV is currently estimated to be between 40-90%; >90% for HHV-6a and b combined; and >80% for HHV-7 (Howley et al., 2021). Infection for all betaherpesviruses is systemic, infecting multiple organs, including the hematopoietic compartments (bone marrow, lungs, liver, and kidneys) and mucosal tissue and brain [reviewed in (Di Luca et al., 1996; Britt, 2007; Santpere et al., 2020)]. In contrast, each virus has specific cellular targets *in vivo* and *in vitro*, especially for latency (discussed below). Direct infection and production of infectious progeny virus during lytic infection generally results in destruction of the infected cell, while latency results in quiescent virus and long-term maintenance for lifelong viral persistence [reviewed in (Mori and Yamanishi, 2007; Goodrum, 2022)].

Latency is a complex and multifactorial process involving viral genome maintenance, viral persistence, cellular control, and immune regulation. Betaherpesviruses are shed in saliva, urine, or genital secretions as frequently as daily even in the presence of neutralizing antibodies and strong cellular immunity, indicating that latency is a dynamic process and suggesting frequent, but specific reactivation events and complex avoidance of the host immune system (Cohen, 2020).

## 5 Betaherpesvirus latency and hematopoiesis

As discussed below, viral infection of HS/HPCs leads to a diverse set of outcomes. Direct infection from diverse viral families, including herpesviruses may adversely affect the HPC pool and neighboring cells. Alteration of the cytokine and cellular transcription factors critical for stem cell maintenance perturbs the HSC pool and immune system maintenance by altering proliferation and differentiation. Prevention of apoptosis or triggering inappropriate differentiation can lead to disease. Alternatively, direct induction of cytolysis leads to progenitor cell destruction, and both direct and indirect effects on neighboring cells leads to immunosuppression. Infected HPCs also serve as a mechanism for viral dissemination including within and between hosts.

### 5.1 HCMV

Viral infection in the bone marrow and the concept that viruses can manipulate this compartment through infection, latency establishment, and immune modulation was first demonstrated in studies of human cytomegalovirus (HCMV). HCMV is the prototypical betaherpesvirus and although most HCMV infections are asymptomatic in healthy individuals (Nogalski et al., 2014), the virus is the leading cause of congenital abnormalities following fetal infection (Voigt et al., 2016; Xia et al., 2021) and is a significant cause of morbidity and mortality during hematopoietic stem cell (Stern et al., 2019; Annaloro et al., 2021) and solid organ (Ramanan and Razonable, 2013) transplant. Transplant-associated HCMV disease results from viral latency-induced myelosuppression and/or acute CMV disease following reactivation (Nogalski et al., 2014; Kotton et al., 2022). It is well established that HCMV establishes latency in HS/HPCs and persists in myeloid lineage cells and that while these cells are required for viral survival and replication, infection also results in myelosuppression (Goodrum, 2022). This apparent conflict of interest on the part of the virus supports the hypothesis that viruses directly and specifically control stem and immune cell fate for evolutionary and survival advantages.

Herpesvirus latency in general, and HCMV latency specifically, is defined as the ability of the virus to enter a cell and maintain the viral genome, without producing infectious virus. While HCMV can infect a wide number of cell types (as demonstrated through a variety of both experimental and *ex vivo* approaches), viral persistence and latency occur in cells of the myeloid lineage. CD34^+^ HPCs provide a critical reservoir of latent HCMV infection (Mendelson et al., 1996; Goodrum et al., 2004) and infection of HPCs contributes to the hematopoietic abnormalities observed in transplant patients (Reeves and Sinclair, 2008; Ljungman et al., 2011; Goodrum, 2016; Safdar and Armstrong, 2019). *In vivo*, latently infected HPCs exit the bone marrow in response to cytokine/growth factor signaling, traffic to the periphery, and differentiate into monocytes and tissue macrophages (Smith et al., 2004; Chan et al., 2008; Goodrum, 2016). This is supported by recovery of infectious virus after allogeneic ex vivo stimulation of peripheral blood monocytes from seropositive patients (Soderberg-Naucler et al., 1997). More recent data suggests unique transcriptional programs in different myeloid lineage cells (Schwartz and Stern-Ginossar, 2019) and differentiation provides a cellular environment appropriate for viral replication and reactivation (Soderberg-Naucler et al., 2001; Smith et al., 2004; Chan et al., 2012a). In parallel, direct infection of monocytes promotes differentiation towards macrophages (Ibanez et al., 1991; Soderberg-Naucler et al., 2001; Smith et al., 2004; Chan et al., 2012b) and while the specific role of monocytes are outside the scope of this review, they are discussed recently elsewhere [reviewed in (Min et al., 2020)]. We have also previously shown that infection of HPCs specifically alters differentiation both *in vitro* and *in vivo* (Crawford et al., 2018; Crawford et al., 2019; Crawford et al., 2020), highlighting the cell type-specific interactions between the virus and host, particularly as related to cellular differentiation. In short, HPCs provide the latent reservoir, monocytes disseminate the virus, and macrophages produce infectious virus for spread; and the cellular differentiation state and cellular heterogeneity play critical roles in this balance.

Previous advances in model systems including development of an *in vitro* latency and reactivation model using primary HPCs (Goodrum et al., 2002; Umashankar and Goodrum, 2014) allowed identification of some of the viral genes and host pathways involved in latency and reactivation [reviewed in (Collins-McMillen et al., 2018)] and description of some of the mechanisms HCMV uses to manipulate the cell. Consistent with established biological differences in different cell types, including source and subtype of the stem cells, functional differences in experimental model systems support differing utility and design strategies to understand the principles and mechanisms of latency [reviewed in (Crawford et al., 2022)].

The evidence for direct infection of HPCs with HCMV is well established, including in CD34^+^ HPCs isolated from adult (bone marrow) and immature cells (cord blood or fetal liver). Infection of HPCs with clinical viral strains results in direct myelosuppressive effects (Maciejewski et al., 1992; Torok-Storb et al., 1992; Movassagh et al., 1996; Sindre et al., 1996; Zhuravskaya et al., 1997; Steffens et al., 1998). These early results have since been replicated by numerous other groups. Additional research also demonstrated that multiple CD34^+^ HPCs subtypes, refined using cellular surface marker expression to reflect stem cell heterogeneity, can be infected with HCMV and that these populations respond differently to infection, including myelosuppressive effects and their ability to support latency (discussed below) (Goodrum et al., 2004; Crawford et al., 2021). These studies demonstrate direct viral inhibition of stem cell maintenance and myelopoiesis, however clinical evidence of global engraftment suppression (Holmberg et al., 1999; Ljungman et al., 2011), suggests HCMV may have a larger role. Indeed, HCMV infection of HPCs or stromal cells results in the modulation of hematopoietic cytokines including IL-6, MIP-1α, and TGF-β, (Apperley et al., 1989; Simmons et al., 1990; Lagneaux et al., 1996; Taichman et al., 1997; Randolph-Habecker et al., 2002; Hancock et al., 2020a). In addition, in murine CMV bone marrow engraftment models, infection is also associated with cytokine dysregulation (Steffens et al., 1998). Further, engraftment of humanized mice with a pool of HPCs wherein only a subset are HCMV-infected is sufficient to result in engraftment delay and suppression (Crawford et al., 2020), which is comparable to clinical patients who receive a seropositive, but undetectable viral load, bone marrow transplant.

### 5.2 HCMV control of latency and cellular properties

How HCMV regulates latency and reactivation and concurrent host cell signaling is not linear or straightforward. Regulation is complex, finely regulated, and dependent both upon the cell type and viral lifecycle phase. HPCs are a critical reservoir for the virus following primary infection both *in vitro* and *in vivo*, likely due in part to the quiescent nature of the cell (reduced cellular proliferation improves viral genome maintenance) and the immune privileged nature of the bone marrow (reduced immune system clearance and improved viral persistence). A summary of viral gene products and host factors with direct links to latency and/or reactivation specifically in HPCs are presented in **Table 1**, although this review will cover these only briefly, as much of this research has been recently and admirably reviewed elsewhere (Elder et al., 2019; Cohen, 2020; Dooley and O’Connor, 2020; Mlera et al., 2020; Poole and Sinclair, 2020; Diggins et al., 2021b; Smith et al., 2021; Goodrum, 2022).

**Table 1:** HCMV and cellular host factors controlling latency in HPCs.

Gene region	HPC Source					Cell Line				Reference
	Embryonic	Fetal liver	Cord blood	Bone marrow	mPB	Kasumi-3	KG-1	RS4;11	in vivo	
Viral Genes										
UL7										Crawford et al., 2018
										Hancock et al., 2021
										Crawford et al., 2021
UL111A										Poole et al., 2014
UL122/UL123										
promoters										Collins-McMillen et al., 2019
										Hale et al., 2020
IE1										Saffert et al., 2010
IE1x4										Tarrant-Elorza et al., 2014
UL133										Umashankar et al., 2011
										Petrucelli et al., 2012
UL135										Umashankar et al., 2014
										Buehler et al., 2016
										Rak et al., 2018
UL136										Caviness et al., 2014
										Caviness et al., 2016
UL138										Petrucelli et al., 2009
										Umashankar et al., 2011
										Petrucelli et al., 2012
										Lee et al., 2015; Lee et al., 2016
										Buehler et al., 2016
US28										Humby and O’Connor, 2015
										Crawford et al., 2019
										Krishna et al., 2019; Krishna et al., 2020; Krishna et al., 2022
LUNA										Poole et al., 2018
Viral miRNAs										
miR-US5-1										Hancock et al., 2021
miR-US5-2										Hancock et al., 2020a; Hancock et al., 2020b
miR-US22										Mikell et al., 2019
miR-US25-1										Diggins et al., 2021a
miR-UL22A										Hancock et al., 2020a
miR-UL112										Hancock et al., 2021
miR-UL148D										Lau et al., 2016
										Pan et al., 2016
Cellular Genes										
AP-1										Krishna et al., 2020
EGFR										Kim et al., 2017
										Rak et al., 2018
EGR-1										Buehler et al., 2016
										Buehler et al., 2019
										Mikell et al., 2019
FOXO										Hale et al., 2020
IFI16										Elder et al., 2019
IL-10										Poole et al., 2015
KAP-1										Rauwel et al., 2015
miRNAs										Poole et al., 2011
SAMHD1										Kim et al., 2019
TGF-β										Hancock et al., 2020a
TNF-α										Forte et al., 2018
Transcriptomic Profiling										
Bulk										Goodrum et al., 2002; Goodrum et al., 2007
										Rossetto et al., 2013
										Cheng et al., 2017
										Forte et al., 2021
Single Cell										Shnayder et al., 2018
miRNAs										Lau et al., 2016
										Mikell et al., 2019

[References for Table 1: (Goodrum et al., 2002; Goodrum et al., 2007; Petrucelli et al., 2009; Poole et al., 2011; Umashankar et al., 2011; Petrucelli et al., 2012; Rossetto et al., 2013; Caviness et al., 2014; Poole et al., 2014; Tarrant-Elorza et al., 2014; Umashankar et al., 2014; Humby and O'Connor, 2015; Lee et al., 2015; Poole et al., 2015; Rauwel et al., 2015; Buehler et al., 2016; Lau et al., 2016; Lee et al., 2016; Pan et al., 2016; Cheng et al., 2017; Kim et al., 2017; Krishna et al., 2017; Crawford et al., 2018; Forte et al., 2018; Poole et al., 2018; Rak et al., 2018; Shnayder et al., 2018; Buehler et al., 2019; Collins-McMillen et al., 2019; Crawford et al., 2019; Elder et al., 2019; Kim et al., 2019; Krishna et al., 2019; Mikell et al., 2019; Hale et al., 2020; Hancock et al., 2020a; Krishna et al., 2020; Crawford et al., 2021; Diggins et al., 2021a; Forte et al., 2021; Hancock et al., 2021; Krishna et al., 2022)].

Latency is characterized by restriction of viral gene expression, including reduction of expression from the major immediate early promoter (MIEP), which normally directs lytic replication through control of the viral immediate early (IE) genes (Mocarski et al., 1996; Marchini et al., 2001). While a decrease in IE expression then appears to a be straightforward measure of latency initiation, herpesviruses are not straightforward. Exon 4 of IE1 has been shown to be actively expressed in HPCs to interact with viral DNA terminal repeats as a potential viral genome maintenance mechanism (Tarrant-Elorza et al., 2014). Early studies to detect viral transcription in experimental latency systems indicated that limited viral genes (the latency factors: US28, UL138, LUNA, UL111A) and long noncoding RNAs (lncRNAs) are expressed (Reeves and Sinclair, 2013; Rossetto et al., 2013; Collins-McMillen et al., 2018). However, more recent studies with newer sequencing methods, including single-cell sequencing, propose that HCMV latency may instead be a pattern of typically lytic gene expression plus lncRNAs (Shnayder et al., 2018). Additional work from the same authors also demonstrates that in both monocytes and HS/HPCs, specific subsets of cells harbor higher viral transcript levels and that these transcriptional programs correlate with latent virus driving cellular differentiation to specific monocyte lineage(s) (Shnayder et al., 2020). Other studies using sensitive qPCR for individual genes of interest also detected gene expression of a variety of transcripts and miRNAs in a variety of latency models (summarized in **Table 1**) and discussed below. These data suggest that latency cannot be measured solely by a transcriptional profile, at least not yet. Combining transcriptional analysis, with genome maintenance, and the ability to productively reactivate following stimulus provides both an internal measure of a latency snapshot timepoint and functional evidence of latency. Importantly, since cells with roles in latency and persistence have unique biological outcomes depending on the viral lifecycle, cellular differentiation stages, and transcriptional programming (Goodrum et al., 2004; Schwartz and Stern-Ginossar, 2019; Min et al., 2020; Crawford et al., 2021) the context of cell heterogeneity and differentiation state must be considered alongside the traditional latency programing factors.

**Figure 2:**
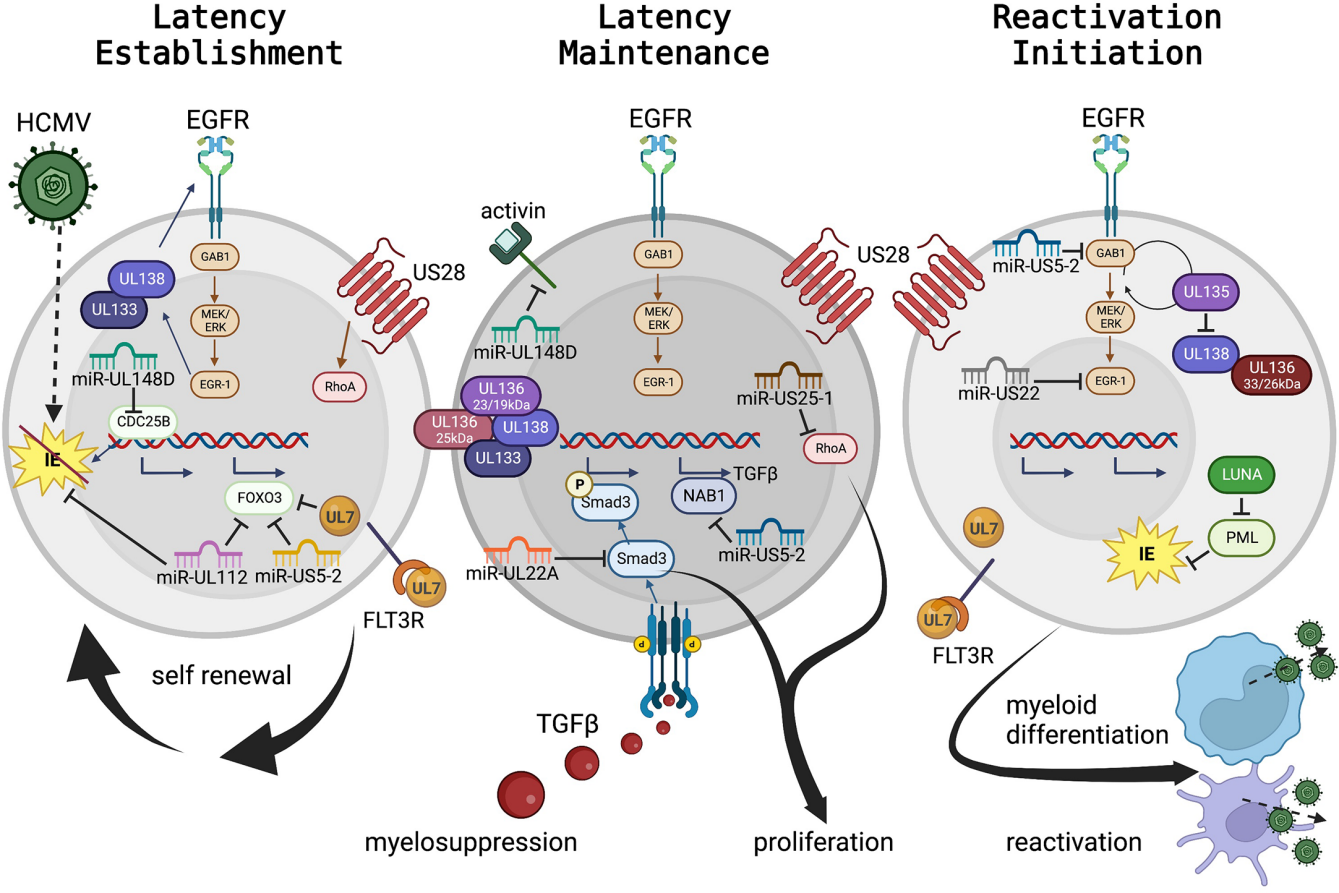
HCMV regulation of progenitor cell mechanisms for latency maintenance, latency establishment, and the initiation of reactivation. HCMV viral proteins and miRNAs controlling cellular signaling, cytokine feedback loops, and as mimics of cellular receptors and ligands to control cell fate, are presented in **Table 1**, and select highlights are discussed here and in the text. Panel 1: Hematopoietic stem and progenitor cells (HS/HPCs) are susceptible to and the preferential site of HCMV latency establishment and maintenance. Specific viral programs regulate entry and genome delivery to the nucleus. Further viral control, including regulation of cellular receptors (including EGFR) by the UL133-UL138 genes control cellular functions (e.g. quiescence, proliferation). Additional viral products regulate signaling pathways, such as US28 regulation of RhoA and UL7 interaction with FLT3R. The viral miRNAs serve as negative regulators of cellular transcriptional pathways to control cellular genes for essential functions (e.g., miR-US5-2 and miR-UL112 regulation of FOXO3 to prevent apoptosis or miR-UL148D regulation of CDC25B to control cell cycle). Viral miRNAs also regulate viral transcription including miR-UL112 inhibition of IE to establish latency. Panel 2: Latency maintenance is also controlled by complex interactions of viral genes, proteins, miRNAs, and cellular pathways including the regulation of proliferation, cell cycle control, and cytokine production (discussed in the text and highlighted here). Latency maintenance is also accompanied by the control of the HPC extracellular environment, mediated in part by viral miRNA control of the master cell regulating cytokine, TGF-β. Panel 3: Viral reactivation and cellular differentiation to the mature myeloid lineages is a quintessential chicken and egg question – which came first? Regardless, reactivation and differentiation are inextricably linked. Viral regulation by the UL133-UL138 region triggers a shift from latency to reactivation by fine-tuning the EGFR pathway. Latency maintenance is relieved, and reactivation and differentiation are initiated by expression of specific viral proteins including US28, UL7 and LUNA; while the viral miRNAs continue to control cellular and viral transcription to regulate differentiation and proliferation which results in the production of new virus particles from mature myeloid lineage cells. (To represent the biological differences of stem cell subsets along myeloid differentiation, nuclear:cytoplasmic ratios are approximately to scale, but some cellular protein localization is solely descriptive rather than representative.).

Latency requires a coordinated regulation of entry, gene expression, genome maintenance, modulation of host responses, and reactivation (Goodrum, 2016). Virus infection (**Figure 2**, left panel) begins with viral entry into the cell stimulating cellular responses. In HPCs, this stimulates EGFR, previously identified as an important factor in viral infection for a variety of other viruses (Zheng et al., 2014) and a key cellular regulator, including of hematopoietic control (Hynes and Lane, 2005; Ryan et al., 2010; Doan et al., 2013). Following transit of the viral capsid to the nucleus, the viral genome is released and is chromatinized (Reeves et al., 2005; Ioudinkova et al., 2006). Genome transcriptional repression is also regulated both by direct repression of IE gene expression (Martínez et al., 2014), expression of IE regulatory proteins [UL138 (Lee et al., 2016)], and through sequestration of transcriptional regulators including pp71 (Kalejta et al., 2003), although the mechanisms are not clearly understood.

Both maintenance of latency (**Figure 2**, middle panel) and reactivation (**Figure 2**, right panel) are regulated by the coordinated effort of viral and cellular control. Cellular regulation, including modulation of all major cellular functions (host cell signaling, proliferation, differentiation, survival, and immune evasion) are controlled by viral signaling and in turn regulate the viral lifecycle. Although much is yet to be discovered, several classes of viral gene products controlling latency and reactivation, and their roles in host control, have been explored.

One master regulator of viral latency and reactivation is the polycistronic UL133-138 locus (Umashankar et al., 2011; Umashankar et al., 2014). This region encodes genes with specific roles in both latency and reactivation [reviewed (Mlera et al., 2020)]. Briefly, following virus entry and stimulation of the MEK/ERK signaling pathway, EGR-1 stimulation in turn stimulates UL138 expression to promote latency establishment and suppress virus replication. EGF receptor recycling is regulated by UL138 to preserve surface expression, control cellular proliferation and differentiation, and promote latency (Buehler et al., 2016). UL133 and UL136p23/19 are also pro-latency proteins, while UL136p25 is context dependent. Temporal regulation of the UL133-UL138 region shifts with cell state (differentiation and proliferation status) and viral kinetics. Expression of UL136p33 and UL136p26 may mediate the shift towards a UL135-dominant state and reduction of UL138’s suppressive effects, which results in a virus replication-promoting cell state. UL135 also promotes EGFR recycling, reverses the effects of UL138, promoting differentiation and triggering reactivation (Buehler et al., 2016). In HPCs the UL136 isoforms are also expressed temporally and work as antagonists to balance these stages (Caviness et al., 2016).

A key example of the differing roles of viral regulation in different cell types, even in the context of the same viral lifecycle phase is US28. During natural infection, US28 is expressed in PBMCs (Patterson et al., 1998), and has been detected in a variety of HPC and monocyte latency models by different groups (Zipeto et al., 1999; Beisser et al., 2001; Goodrum et al., 2002; Cheung et al., 2006; Humby and O'Connor, 2015; Cheng et al., 2017). US28 is a viral G protein-coupled receptor that regulates viral latency establishment and reactivation, and controls cellular signaling pathways during entry and reactivation. In CD34^+^ HPCs, US28 constitutive signaling is required for latency establishment and virus-mediated reprogramming of infected cells (Humby and O'Connor, 2015; Krishna et al., 2017; Krishna et al., 2019); yet in HPCs and in humanized mice US28 ligand binding activity is required for latency and reactivation (Crawford et al., 2019). The apparent difference in these outcomes likely highlights the fine-tuned control of *different* hematopoietic cells (including distinct HS/HPC subsets) by HCMV. These data clearly demonstrate a key role for US28 regulation of the host cell and the control of latent *vs* reactivation states, although further understanding of the specific mechanisms and distinct roles in different cell types are still needed.

Part of latency regulation is control of host cell signaling. Fine tuning of cellular (and viral) signaling pathways can be accomplished through miRNA targeting of transcripts. HCMV encodes 22 mature miRNAs with diverse roles at all stages of viral infection [reviewed (Diggins et al., 2021b)], including in experimental latency models and in patient samples (Zhou et al., 2020). During latency, select miRNAs regulate maintenance, reactivation, and cellular control in HPCs in conjunction with viral and cellular proteins. In addition, miRNAs also directly regulate cell signaling in HPCs. miR-UL148D is expressed early in HPC infection (Pan et al., 2016) and during latency (Mikell et al., 2019). Like US28, miR-UL148D has potentially contradictory roles in regulating latency and reactivation, both by promoting latency establishment by downregulating an IE activator (Pan et al., 2016) and/or blocking cellular activin receptor activity to decrease IL-6 secretion during latency (Lau et al., 2016). More recent studies also demonstrate roles for miRNAs to control cell proliferation through regulation of the RhoA signaling axis by miR-US25-1 (Diggins et al., 2021a) or in coordination with UL135 and UL138 to regulate EGFR by miR-US22’s regulation of EGR-1 (Mikell et al., 2019). In addition, miR-US5-1 and miR-UL112 work in concert with UL7 to protect cells from apoptosis (Hancock et al., 2021) and promote HPC survival.

HCMV also regulates global cellular cytokines to control both direct cellular effects (including latency, reactivation, and cell control) and indirect effects (altering neighboring cells, including the induction of myelosuppression). miR-US5-2 and miR-UL22A work in concert to stimulate TGF-β expression (through miR-US5-2 downregulation of NAB1) and simultaneous downregulation of SMAD3 by miR-UL22A (Buehler et al., 2019; Mikell et al., 2019; Hancock et al., 2020a). By this mechanism, the latently infected cell can produce TGF-β to regulate the external environment, resulting in myelosuppression of uninfected neighboring cells, and yet protect the host HPC from the negative effects of TGF-β.

Viral reactivation can be triggered by global stimulation, including through cytokine manipulation and direct genetic or protein level control. G-CSF mobilization of stem cells into the periphery in patients stimulates viral reactivation and disease (Anderson et al., 2003) and can be used as a robust trigger for viral reactivation in humanized mouse models both with and without functional HCMV-specific immune responses (Smith et al., 2010; Crawford et al., 2017). In addition to its early effects, specific regulation of the EGFR pathway by viral miRNAs (miR-US5-2 and miR-US22) and viral proteins (UL135 and UL138) also control reactivation (Hancock et al., 2020a; Hancock et al., 2020b). Other viral proteins, such as UL7, function as homologs to cellular receptors to control differentiation and stimulate reactivation (Crawford et al., 2018). Control of differentiation from an HPC to the monocytic lineage, either through directed pathways or more global effects such as G-CSF stimulation, is a well-established trigger for reactivation (Sinclair and Reeves, 2014). Crosstalk between these pathways and other cellular signaling pathways are expected from our understanding of cell biology, and additional evidence indicates that other viral proteins (including US28) also contribute to this signaling although how these viral factors synergize for control are not yet known.

Although these studies clearly demonstrate the intricate virus-cell interactions that regulate the host and allow viral persistence, much remains to be defined about the central strategies of latency, especially in context of stem cell subsets.

### 5.3 HHV-6a and HHV-6b

HHV-6 (both HHV-6a and HHV-6b) has many commonalities with HCMV. Infection is chronic and widespread, and these viruses are genetically the most closely related betaherpesviruses. Cell tropism of HHV-6 is different however, as primary and replicative infection occurs not only in monocytes, but also B-cells, NK cells, neural cells, and with a primary preference for CD4^+^ T-cells and persistence in salivary glands (Cohen, 2020). *In vitro* infection of CD34^+^ HPCs with HHV-6 suppresses hematopoietic colony formation of granulocyte-macrophage, erythroid, and megakaryocyte lineages (Isomura et al., 2003). Interestingly, while both HHV-6a and HHV-6b demonstrate an MOI-dependent general effect on *in vitro* colony formation and on erythroid lineage colonies specifically, only HHV-6b has a significant suppressive effect on granulocyte-macrophage lineage differentiation (Isomura et al., 1997). The difference in lineage-specific differentiation outcomes following infection of HHV-6a (erythroid only) compared with the broader myelosuppressive effects of HHV-6b and HCMV support a role for unique viral-cell interactions specific to each virus.

Data from stem cell transplant recipients demonstrates clear viral replication and/or reactivation (greater than 10^3^ copies/10^6^ PBMCs, compared to undetectable viral load in immunocompetent individuals) (Boutolleau et al., 2003). In this study, viral load was also higher in patients with engraftment complications (including delayed neutrophil engraftment, graft *vs* host disease, and/or overt viral disease). *In vitro* analysis, again using CD34^+^ HPCs with specific differentiation conditions for megakaryocyte *vs* non-megakaryocyte colonies, demonstrated that HHV-6a and HHV-6b infection inhibit global colony formation regardless of the presence of supportive serum or additional thrombopoietin (TPO) when used to support HSC maintenance and megakaryocyte differentiation in the culture (Isomura et al., 2000).

Similar to HCMV, HHV-6 genomic DNA can be detected in HPCs (Isomura et al., 1997), however, in contrast, heat-inactivation and/or use of supernatants from HHV-6 infected cells abolishes the suppressive effects on HPCs (Isomura et al., 2000), strongly supporting a majority direct effect of HHV-6 on the suppression of HPC differentiation rather than indirect effects on the bone marrow environment. Once infected however, HPCs support early expression of IE genes followed by later downregulation of transcription in both early and late progenitors (Isomura et al., 2003), suggesting the potential for latency establishment, although this has not yet been demonstrated *in vitro*.

Unique out of all herpesviruses, HHV-6 infection also results in viral integration in 1-2% of healthy individuals (Arbuckle et al., 2010; Morissette and Flamand, 2010). While integration is usually near the subtelomeric/telomeric junction on the chromosome, and is rarely oncogenic due to this location, this virus can be transmitted through the germline and the long-term effects of herpesvirus integration here are still unknown [reviewed (Pantry and Medveczky, 2017)]. There is also little known about the mechanisms of latency and reactivation involving integrated HHV-6, although the involvement of telomerase and the lack of a consistent integration site leave room for speculation as to how these unique viral mechanisms regulate host cell control. Evidence from other studies, including from HIV (Pasternak and Berkhout, 2023), HPV (McBride and Warburton, 2017), and viral vector integration specifically in stem cells (Kimbrel and Lanza, 2020), support a clear role for viral integration, that when combined with long latency periods, such as with integrated HHV-6, can result in disease.

While less is known about the regulation of HHV-6 latency, some commonalities have been established. The HHV-6 IE2 protein shares structural homology with EBNA1 (EBV) and LANA (KSHV), suggesting that it may also be involved with genome maintenance by host chromosome tethering (Nishimura et al., 2017), although no functional studies have yet been performed. In integrated HHV-6 infection, the genome is enriched with heterochromatin (Saviola et al., 2019) which is consistent with genome silencing required for latency maintenance. Similar to HCMV, the frequency of infection is low (1:10,000 to 1:100,000), hampering gene expression analysis. Although HHV-6 encodes four latency-associated transcripts in macrophages (Kondo et al., 2002) and an U95 RNA has also been detected in healthy donor PBMCs (Rotola et al., 1998), no studies have assessed viral transcription in HPCs. HHV-6 also encodes miRNAs expressed during lytic replication (Tuddenham et al., 2012), including one that targets the IE gene U86 (Nukui et al., 2015), which may suggest a role in early reactivation, although the function of these miRNAs during latency has yet to be determined.

### 5.4 HHV-7

The final human betaherpesvirus, HHV-7 is understudied. Prior work demonstrated that like the other betaherpesviruses, HHV-7 is a common and chronic human infection. HHV-7 infects T-cells, epithelial cells of the lungs, and salivary glands, with latency establishment in CD4^+^ T-cells (Cohen, 2020). Little is known about the molecular mechanisms of viral gene regulation, or a role in progenitor cells, although HHV-7 DNA has been detected in CD34^+^ HPCs from bone marrow (Mirandola et al., 2000).

To determine if HHV-7 also governs hematopoietic progenitor cell fate and/or plays a role in transplant outcomes, several studies assessed the role of direct HHV-7 infection in HPCs, although these data are contradictory. In the same study as discussed above for HHV-6 (Boutolleau et al., 2003), where increasing HHV-6 viral loads correlated with immunosuppression and adverse outcomes, HHV-7 viral loads were comparable in transplant patients and healthy controls, although the authors suggest based on kinetic viral load data that HHV-7 may act as a cofactor of HHV-6 reactivation. In contrast, in a cohort study of pediatric patients which included allogenic and autologous bone marrow transplants, while less than 6% of patients had detectable HHV-7 viral loads, all HHV-7+ patients also had complications including graft *vs* host disease and/or co-infections (Khanani et al., 2007).

To determine if infection of progenitor cells alters hematopoietic colony formation, several studies assessed the *in vitro* infection of CD34^+^ HPCs with HHV-7. The first study found that HHV-7 infection had no effect on hematopoietic colony formation regardless of differentiation outcome (Isomura et al., 1997). Later studies also verified that HHV-7 infection also had no influence on differentiation either in the presence or absence of TPO (Isomura et al., 2000). However, an additional study found that HHV-7 infection of cord blood HPCs slightly altered the proportions of granulocytic/macrophagic and erythroid colony formation and significantly inhibited pluripotent colony formation (Mirandola et al., 2000). This study also demonstrated that this effect resulted from direct infection of HPCs, as neutralizing serum blocked changes in colony formation following HHV-7 infection. Interestingly, similar to HCMV, HHV-7 also has a seemingly contradictory role of both inhibiting hematopoiesis and directly stimulating differentiation of certain cell types. HHV-7 infection increases myeloid but not erythroid maturation in liquid culture yet viral mRNA is maintained in both cell types (Mirandola et al., 2000), providing additional evidence that the viral life cycle and cell type-specific functions are intertwined.

## 6 Discussion and perspectives

Viral latency, in betaherpesviruses and others, is regulated by a complex system of viral and cellular control. During latency, the virus promotes preferential maintenance of the viral genome, increased host cell survival, and manipulates the cellular environment, including suppression of neighboring cells and immune control. The idea that HCMV latency is dynamic with the virus responding specifically to inter- and intra-host signals was recently reviewed and highlights the complexity of virus-host interactions (Goodrum, 2022). This is especially relevant in the context of betaherpesvirus infection in hematopoietic progenitor cells. HCMV, HHV-6, and HHV-7 are master regulators of cells, controlling cellular signaling, responses, and fate through a variety of mechanisms that are temporally regulated to match the viral lifecycle and the cell type and differentiation stage. In HCMV, multiple viral proteins (Collins-McMillen et al., 2018), miRNAs (Chen et al., 2022; Diggins and Hancock, 2022), and cellular pathways (Smith et al., 2021) regulate viral and host cell signaling (**Figure 2**). This regulation varies depending on the cell type or model system hosting the virus (Crawford et al., 2022) and is specific to cell fate and viral lifecycle stage, setting up precisely tuned regulatory mechanisms by the virus. While fewer specific details are known about the role of HHV-6 or HHV-7 proteins in latency and cellular control, betaherpesvirus infection has implications in complex disease, including during transplant (Higdon et al., 2023) and latent viral proteins have promise as novel therapeutics (Perera et al., 2021; Berg and Rosenkilde, 2023).

Understanding the complexity of latency also necessitates understanding the specifics of the host cell – which for betaherpesviruses are unique populations of hematopoietic stem and progenitor cells and their individual properties. Currently, stemness is defined by the ability to self-renew and the absence of lineage-specific programing. More recent data suggests that HSCs are more tightly defined by unique transcriptomic, metabolomic, and cellular properties that are not (yet) clearly linked with function or surface marker “definitions” (Laurenti and Göttgens, 2018). This is supported by transcriptome data from the Human Protein Atlas which shows that 61% of *all* human genes are expressed in the bone marrow and more than 10% have increased expression here compared to other tissue types (Uhlén et al., 2015). This evidence of stem cell signatures and the preference for betaherpesvirus establishment of latency in these cells sets up the premise of a functional interplay between virus and host. Both HCMV and HHV-6 have differential effects in HPCs of different identity (Isomura et al., 2003; Goodrum et al., 2004; Crawford et al., 2021), and HCMV and HHV-7 also have seemingly contradictory roles by both suppressing global hematopoiesis yet activating specific hematopoietic lineages (Mirandola et al., 2000; Goodrum, 2022). Understanding this interplay has significant implications for basic viral and stem cell biology, and for new therapeutic tools for stem cell function and chronic viral infection, and further research is required to understand the complex players involved.

## Author contributions

The author confirms being the sole contributor of this work and has approved it for publication.
